# Divergent Mechanisms Activating RAS and Small GTPases Through Post-translational Modification

**DOI:** 10.3389/fmolb.2021.707439

**Published:** 2021-07-08

**Authors:** Natsuki Osaka, Yoshihisa Hirota, Doshun Ito, Yoshiki Ikeda, Ryo Kamata, Yuki Fujii, Venkat R. Chirasani, Sharon L. Campbell, Koh Takeuchi, Toshiya Senda, Atsuo T. Sasaki

**Affiliations:** ^1^ Institute for Advanced Biosciences, Keio University, Tsuruoka, Japan; ^2^ Division of Hematology and Oncology, Department of Internal Medicine, University of Cincinnati College of Medicine, Cincinnati, OH, United States; ^3^ Department of Bioscience and Engineering, College of Systems Engineering and Science, Shibaura Institute of Technology, Saitama, Japan; ^4^ Structural Biology Research Center, Institute of Materials Structure Science, High Energy Accelerator Research Organization (KEK), Tsukuba, Japan; ^5^ Faculty of Environment and Information Studies, Keio University, Fujisawa, Japan; ^6^ Department of Molecular Genetics, Institute of Biomedical Science, Kansai Medical University, Osaka, Japan; ^7^ Graduate School of Science, Osaka City University, Osaka, Japan; ^8^ Department of Biochemistry and Biophysics and Lineberger Comprehensive Cancer Center, University of North Carolina at Chapel Hill, Chapel Hill, NC, United States; ^9^ Cellular and Molecular Biotechnology Research Institute, National Institute of Advanced Science and Technology, Tokyo, Japan; ^10^ Department of Accelerator Science, School of High Energy Accelerator Science, SOKENDAI (The Graduate University for Advanced Studies), Tsukuba, Japan; ^11^ Faculty of Pure and Applied Sciences, University of Tsukuba, Tsukuba, Japan; ^12^ Department of Cancer Biology, University of Cincinnati College of Medicine, Columbus, OH, United States; ^13^ Department of Neurosurgery, Brain Tumor Center at UC Gardner Neuroscience Institute, Cincinnati, OH, United States

**Keywords:** RAS, post-translational modification, G-domain, ubiquitylation (ubiquitination), lysine modification, cysteine oxydation, cancer, RAS superfamily GTPase

## Abstract

RAS is a founding member of the RAS superfamily of GTPases. These small 21 kDa proteins function as molecular switches to initialize signaling cascades involved in various cellular processes, including gene expression, cell growth, and differentiation. RAS is activated by GTP loading and deactivated upon GTP hydrolysis to GDP. Guanine nucleotide exchange factors (GEFs) and GTPase-activating proteins (GAPs) accelerate GTP loading and hydrolysis, respectively. These accessory proteins play a fundamental role in regulating activities of RAS superfamily small GTPase *via* a conserved guanine binding (G)-domain, which consists of five G motifs. The Switch regions lie within or proximal to the G2 and G3 motifs, and undergo dynamic conformational changes between the GDP-bound “OFF” state and GTP-bound “ON” state. They play an important role in the recognition of regulatory factors (GEFs and GAPs) and effectors. The G4 and G5 motifs are the focus of the present work and lie outside Switch regions. These motifs are responsible for the recognition of the guanine moiety in GTP and GDP, and contain residues that undergo post-translational modifications that underlie new mechanisms of RAS regulation. Post-translational modification within the G4 and G5 motifs activates RAS by populating the GTP-bound “ON” state, either through enhancement of intrinsic guanine nucleotide exchange or impairing GAP-mediated down-regulation. Here, we provide a comprehensive review of post-translational modifications in the RAS G4 and G5 motifs, and describe the role of these modifications in RAS activation as well as potential applications for cancer therapy.

## Introduction

**FIGURE 1:**
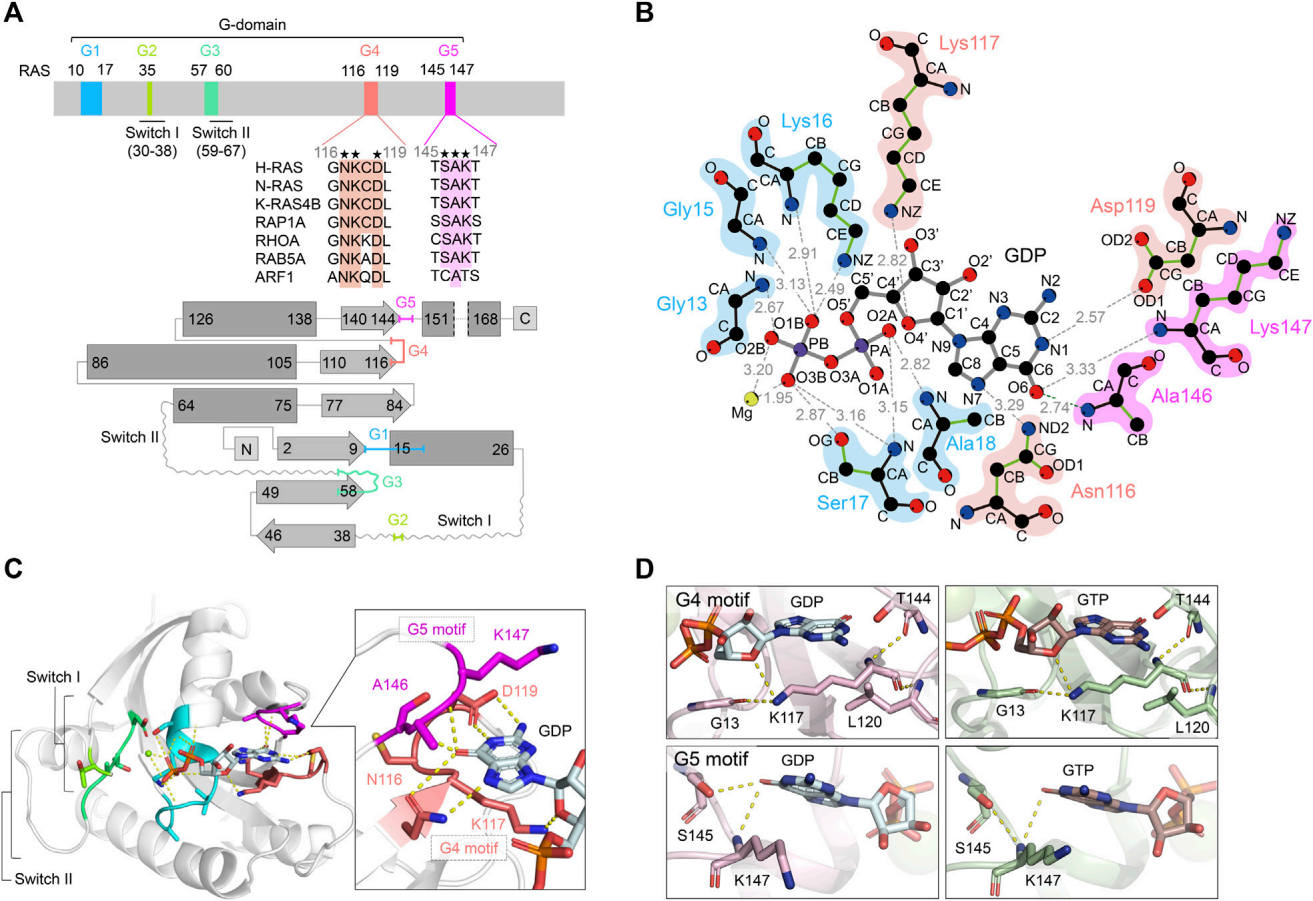
Overview of RAS structures and the guanine nucleotide-dependent interactions of G4 and G5 motifs. **(A)** A schematic diagram of the RAS G-domain. Upper: Multiple sequence alignment of the RAS isotype G4 and G5 motifs and representative RAS superfamily members are shown. Conserved residues are annotated by asterisks. Lower: the secondary structures and topology of RAS. α-helices and β-sheets are shown in rectangle and arrow shape, respectively. Color theme for each G motif (G1: cyan, G2: light green, G3: green, G4: coral pink, G5: magenta) are consistent throughout the figures. **(B)** Interaction of H-RAS G-motifs with GDP (PDB: 4Q21) with hydrogen bonds. The plots were generated by LigPlot (Wallace et al., 1995) and the modified for clarity. The hydrogen bonds are shown in gray dotted lines with the distance between atoms. For amino acid residues, the main chains are shown in black, and the side chains are shown in green. Each atom is shown in a sphere and colored as follows: carbon, black; oxygen, red; nitrogen, blue; phosphorous, purple; magnesium, lime yellow. **(C)** The crystal structure of GDP-bound H-RAS (PDB: 4Q21). The hydrogen bonds are shown as dotted lines and Mg^2+^ ion as a purple sphere. Protein is shown as gray helix and the interacting residues of G4 and G5 motifs with the guanine moiety are shown in licorice representation in the inset. **(D)** Interactions of Lys117 within the G4 motif (upper panels) and Lys147 within the G5 motif (lower panels) with GDP-bound K-RAS (PDB: 6MBT) (left panels) and GTP-bound K-RAS (PDB: 5VQ2) (right panels). Hydrogen bond interactions are shown as dotted lines. Protein is rendered as cartoon and residues interacting with Lys117 or Lys147 are shown in licorice representation.

RAS superfamily small GTPases consist of more than 170 members. They act as molecular switches cycling between GTP-bound “ON”- and GDP-bound “OFF”-states and play a crucial role in transducing signals that direct various cellular activities (Wennerberg et al., 2005). The RAS superfamily and other GTPase families (e.g., heterotrimeric G-proteins, elongation factors) contain a core guanine binding (G)-domain that possesses a Rossman fold. This structural unit enables high-affinity binding to GTP and GDP, as well as the ability to hydrolyze GTP (Figure 1A). RAS proteins have been the subject of intense investigation, as they are the most prevalent oncoprotein in human cancer. In this review, we will focus on the RAS G-protein and introduce a new layer of the regulation by post-translational modifications outside the canonical Switch regions. We will also discuss potential applications for cancer therapy.

## The Overview of RAS Structure and Regulation

### The Conserved G-Motif Is Required for High-Affinity GTP and GDP Binding of RAS

The core G-domain of RAS superfamily small GTPases consists of a six-stranded β-sheet and five α-helices, which contain five functional motifs, G1-G5 motifs (Figures 1A,C; Wennerberg et al., 2005; Wittinghofer and Vetter, 2011). The G1 motif is also referred to as P-loop or Walker A/phosphate-binding loop. The G2 and G3 motifs contain regions termed Switch I and Switch II (collectively referred to as Switch regions). The P-loop and Switch regions form interactions with the β- and γ-phosphate groups of GTP, GDP and Mg^2+^. The Switch regions differ in conformation between the GDP-bound “OFF” state to the GTP-bound “ON” state (Kinoshita et al., 1999; Wittinghofer and Vetter, 2011). The GTP-bound “ON” state is considered the active state as it adopts a conformation that leads to increased affinity for downstream effectors (e.g., RAFs, class I PI3Ks), thereby transmitting signals. For example, the affinity of the GTP-bound RAS for RAF1 (CRAF) is approximately 1000-fold higher than that of GDP-bound RAS (Herrmann et al., 1995; Kiel et al., 2009).

The G4 and G5 motifs—the focus of this review—play a critical role in the high-affinity binding of RAS to GTP and GDP through guanine base and ribose recognition (Vetter and Wittinghofer, 2001; Wittinghofer and Vetter, 2011). In fact, the substitution of Lys117 or Asp119 in the G4 motif significantly reduces guanine ligand binding, leading to greatly enhanced guanine nucleotide dissociation (Feig et al., 1986; Denayer et al., 2008; Baker et al., 2013b). In the RAS superfamily, the G4 motif contains an “N-K-X-D” sequence (X denotes any amino acid, ^116^NKCD^119^ in human RAS) and is a major determinant of guanine nucleotide specificity. The amino acid residues in the G4 motif are strictly conserved, except for the third position (X). In the structure of the GDP-bound RAS, Lys117 in the G4 motif interacts with Gly13 of the G1 motif and the guanine nucleotide ribose sugar (Figures 1B,D). Since Lys117 and Asp119 are highly conserved residues present in the guanine-specificity region of all guanine-nucleotide-binding proteins, mutations at these residues significantly alter the nucleotide exchange rates. Mutations in Lys117 drastically reduce the nucleotide-binding affinity and influence interactions with P-loop residues. As Asp119 makes a key hydrogen bond interaction with the guanine N1 atom (Figures 1B,D; Pai et al., 1989), mutations in Asp119 will also influence nucleotide binding affinity (Cool et al., 1999). The influence of Asp119 mutations on nucleotide-binding affinity is significantly lower than that of Lys117 mutations. The G5 motif has an “S-A-X” sequence (X denotes any amino acid, ^145^SAK^147^ in human RAS), which also interacts with the guanine moiety and is required for selective and high-affinity binding of RAS to guanine nucleotides (Figure 1B). The amino group of Ala146 forms a hydrogen bond with the O6 atom of the guanine ring, and the amino group of Lys147 forms a hydrogen bond with the N2 atom of the guanine ring (Figure 1D; Pai et al., 1989).

### RAS Regulation by GEFs and GAPs

**FIGURE 2:**
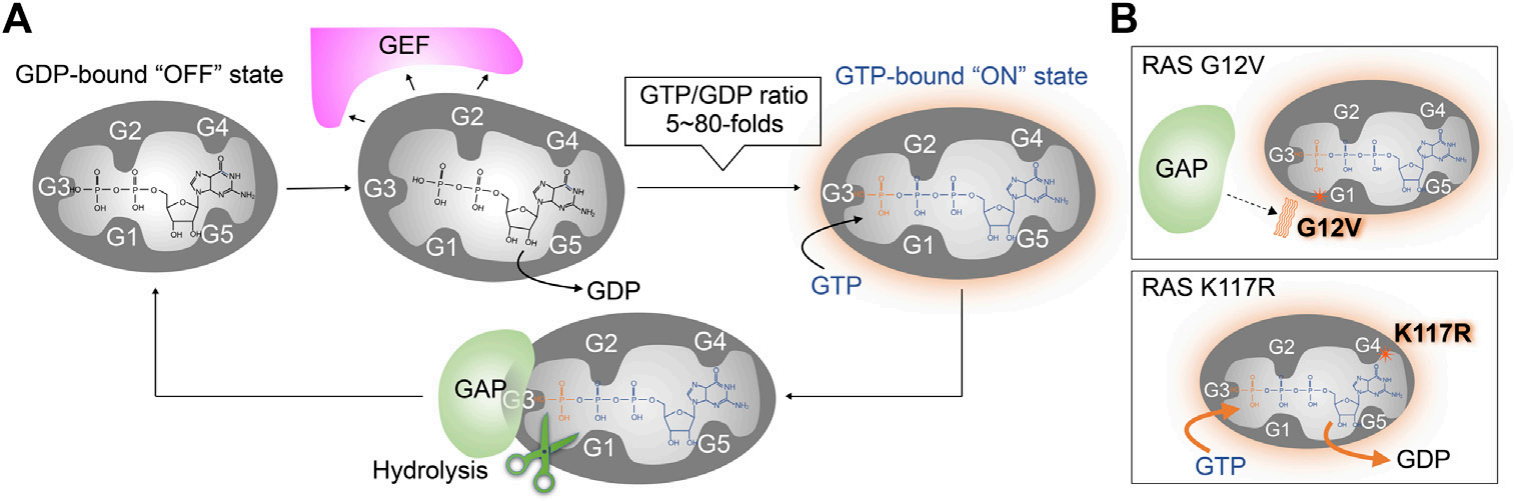
Wild type and oncogenic RAS regulation by GEFs and GAPs. **(A)** The RAS nucleotide cycling regulated by GEF and GAP. GEFs bind to RAS, inducing conformation changes that reduce the RAS affinity for guanine nucleotide ligands. This leads to the dissociation of GDP and the formation of the nucleotide-free apo-form of RAS from the GDP-bound “OFF” state. Stochastic GTP loading to the apo-form of RAS facilitating the GTP-bound “ON” state, due to the higher GTP/GDP ratios in the cell. GAPs bind to the GTP-bound RAS and increases its intrinsic GTPase activity for GTP hydrolysis. **(B)** Activation mechanism of oncogenic RAS mutant. Upper: The RAS G12V oncogenic mutant impairs both intrinsic GTPase activity and GAP-dependent GTP hydrolysis. Lower: the RAS K117R mutant maintains intrinsic GTPase activity and GAP-dependent GTP-hydrolysis, but decreases the nucleotide affinity, leading to an increased GTP/GDP exchange.

In mammalian cells, three families of GEFs and six families of GAPs have been identified that act on RAS (Vigil et al., 2010; Henning et al., 2015; Li et al., 2018; Gray et al., 2020; Stalnecker and Der., 2020). Similarly, there are multiple GEFs and GAPs associated with other RAS superfamily small GTPases (Bos et al., 2007; Cherfils and Zeghouf, 2013). GEFs are regulated by kinase-mediated phosphorylation and interactions with second messengers (e.g., Ca^2+^, diacylglycerol, cAMP), which is often coupled with changes in subcellular localization (Bos et al., 2007; Vigil et al., 2010; Cherfils and Zeghouf, 2013). In unstimulated cells, RAS exists predominately in the GDP-bound “OFF” state. Once the GEF is activated or co-localized with RAS, the GEF binds to RAS and interferes with the RAS/guanine ligand. This leads to the dissociation of GDP from RAS. As the affinity of RAS to GTP and GDP is similar (Feuerstein et al., 1987; John et al., 1993; Ford et al., 2009), the frequency of RAS activation reflects the intracellular GTP/GDP ratio (5∼80 fold) in mammalian cells (Traut, 1994), to promote the population of RAS in the GTP-bound “ON” state *via* a stochastic GTP loading (Figure 2A). RAS is deactivated upon hydrolysis of the phosphate bond between the β- and γ-phosphate of GTP. Although the rate of intrinsic GTP hydrolysis activity is slow, RAS GAPs bind to GTP-bound RAS and stimulate GTP hydrolysis. In the structure of RAS GAPs (p120 RASGAP) and NF1-bound RAS, GAP binding stabilizes the active site and provides an arginine finger, which directly interacts with the β- and γ-phosphate of GTP, to greatly enhance the GTP hydrolysis rate of RAS (Figure 2A; Scheffzek et al., 1997; Kötting et al., 2008).

### Oncogenic Mutation Within the G4 and G5 Motifs

**FIGURE 3:**
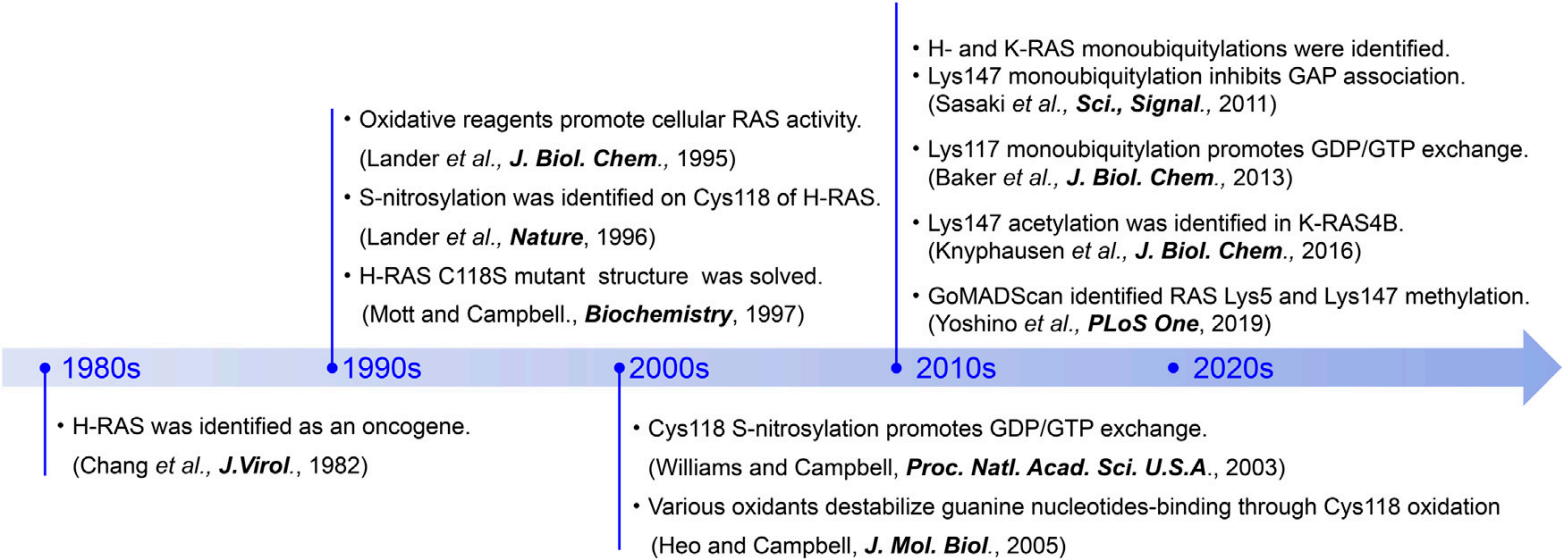
A chronicle of RAS-related discoveries highlighted in this review.

In mammalian cells, there are three isotypes of RAS, named H-RAS, K-RAS, and N-RAS. Single point mutations in RAS that promote constitutive RAS activation and tumorigenesis (Bos, 1989; Downward, 2003; Malumbres and Barbacid, 2003; Karnoub and Weinberg, 2008; Pylayeva-Gupta et al., 2011; Ratner and Miller, 2015) and developmental disorders (Tidyman and Rauen, 2009; Rauen, 2013; Borrie et al., 2017; Simanshu et al., 2017) were first identified in the early 1980s (Figure 3; Chang et al., 1982). These were later found to be present in approximately 25% of human cancers (Forbes et al., 2010; Prior et al., 2012; Prior et al., 2020), and over 100 oncogenic mutations have since been identified in human RAS. Among them, the K-RAS G12C oncogenic mutation is present in about 3–14% of cancer patients (Prior et al., 2012; Prior et al., 2020; Nassar et al., 2021) and has been targeted for drug discovery efforts (Ostrem et al., 2013; Lito et al., 2016; Janes et al., 2018; Hallin et al., 2020; Moore et al., 2020). However, the K-RAS G12C inhibitors do not act on other oncogenic mutants as they lack the reactive cysteine at position 12 needed for covalent ligation and inhibition. Thus, further understanding of RAS regulatory mechanisms is critical to developing new therapeutic approaches for targeting RAS-driven cancers and developmental disorders.

Gly12 and Gly13 in the G1 motif and Gln61 in the G3 motif are known as hot spots for RAS oncogenic mutations (Moore et al., 2020; Prior et al., 2020). One common feature of these mutants is that they are impaired in GTP hydrolysis and thus populated in the GTP-bound “ON” state (Figure 2B upper panel) (Gideon et al., 1992). In addition to the impaired GTP hydrolysis, the G13D and Q61L mutants are unique in that they also display enhanced intrinsic guanine nucleotide exchange (Smith et al., 2013). The improvements in sequencing technology in the 2000s have uncovered additional point mutations in the G4 (e.g., K117N) and G5 (e.g., A146T) motifs (Edkins et al., 2006; Denayer et al., 2008; Wójcik et al., 2008; Smith et al., 2010; Gremer et al., 2011; Niihori et al., 2011) that promote RAS activation.

These oncogenic mutations in the G4 and G5 motifs of RAS retain GTP hydrolytic activity but greatly accelerate the guanine nucleotide exchange rate that renders the GTPase less sensitive to GEF-regulation (Denayer et al., 2008; Janakiraman et al., 2010; Figure 2B lower panel). As indicated in the previous section “The Conserved G-Motif is Required for High-Affinity GTP and GDP Binding of RAS,” a subset of amino acids in the G4 and G5 motifs are highly conserved as they directly interact with the guanine ring and are important for the high affinity and specificity of the guanine nucleotide. For example, even conservative mutations, such as K117N, K117R, and K147R, can significantly increase nucleotide exchange rate and populate RAS in the GTP-bound “ON” state (Sasaki et al., 2011; Figure 2B lower panel). X-ray structural analysis indicates that the guanidium group of Arg117 associated with the K-RAS K117R mutant forms an additional interaction with the amide group of Asn85, resulting in destabilization of key nucleotide ligand interactions with the G4 motif (Lys117) and P-loop (Gly13) (Denayer et al., 2008; Figure 1D). These observations suggest that the conserved amino acids in the G4 and G5 motifs are critical for guanine nucleotide-binding—i.e., perturbations in these key residues may promote RAS activation.

### Post-translational Modifications Outside the Switch Regions

While missense mutations within the key residues in G4 and G5 motifs can promote RAS activation, post-translational modification (PTM) of these residues is yet another mechanism that can alter guanine nucleotide interactions and RAS activity. PTMs of proteins are key regulatory events in many cellular processes. Eukaryotic cells possess a variety of enzymes responsible for PTMs, such as Ser/Thr/Tyr kinases, methyltransferases, acetyltransferases, and ubiquitin ligases. PTMs by these enzymes are dynamic and, in most cases, reversible. It is well-known that the G-domain and C-terminal region of RAS is regulated by various PTMs (Ahearn et al., 2018). Furthermore, accumulating evidence indicates that RAS undergoes S-nitrosylation of select cysteine residues, as well as acetylation, methylation, and ubiquitylation of lysine residues within the G4 and G5 motifs (Lander et al., 1995; Sasaki et al., 2011; Knyphausen et al., 2016; Yoshino et al., 2019; Figure 3). These PTMs can upregulate RAS activity by increasing the guanine nucleotide exchange rate and/or inhibiting GAP-mediated GTP hydrolysis.

## PTM Within the RAS G4 Motif (^116^NKCD^119^)

### S-Oxidation and S-Nitrosylation of Cys118 in the G4 Motif

Cells are often exposed to various stresses, such as increased reactive oxygen species (ROS). ROS are continuously generated through the mitochondrial electron transport chain, peroxidases, xanthine oxidase, lipoxygenase, NADPH oxidases, and heme-enzyme reactions. ROS can be generated by exogenous stimuli, such as UV and ionizing radiation, ethanol intake, oxidized food, metal ion overload (e.g., Fe and Cu), and smoking. Also, nitric oxide (NO) is generated endogenously by nitric oxide synthases (NOS) and exogenously by nitrogen oxides in air pollution (NO_X_) (e.g., car exhaust) and nitro compounds (Davies, 2016).

**FIGURE 4:**
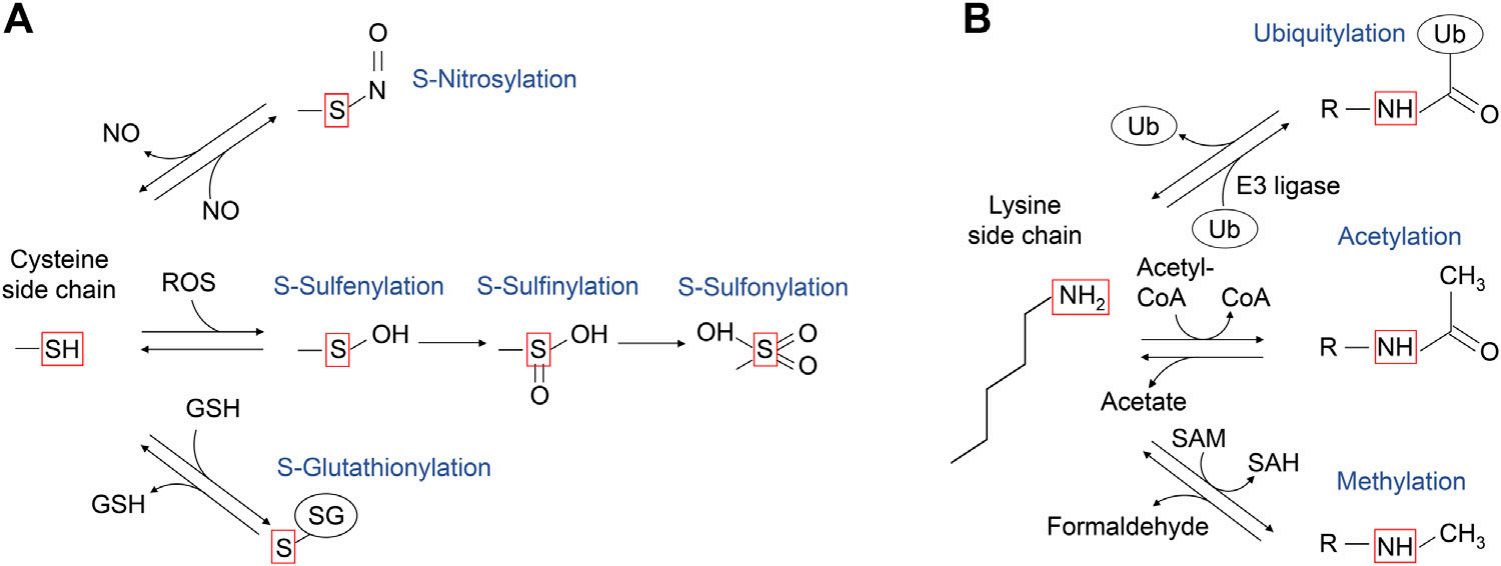
A schematic diagram of the post-translational modifications of cysteine and lysine side chains. **(A)** The sulfur atom of cysteine side chain can undergo several oxidative modifications, including those shown in the red box. S-nitrosylation can be generated upon reaction with nitric oxide (NO). Upon the reaction with reactive oxygen species (ROS), the sulfur atom of cysteine side chain can undergo S-sulfenylation, and further oxidation to S-sulfinic and S-sulfonic states. The cysteine side chain can also form mixed disulfides, including reaction with glutathione (GSH) to undergo reversible S-glutathionylation. **(B)** The ε-amino group of lysine side chain can undergo several modifications as shown in the red box. The portion of modified lysine side chains is shown as “R-NH”. Ubiquitylation is mediated by ubiquitin E3 ligase, while deubiquitylation is mediated by deubiquitylases. Lysine acetyltransferases use acetyl-CoA as the acetyl-donor for lysine acetylation, which can be reversed by acetylated lysine deacetylases. Lysine methyltransferases use S-adenosylmethionine as a methyl donor for lysine methylation, which is reversed by methylated lysine demethylase, coproducing formaldehyde.

Cysteine is a key amino acid in proteins for maintaining redox balance. Cysteine has a reactive thiol side chain (Cys-SH), which can undergo one- and two-electron oxidation reactions. Also, cysteine can undergo several reversible oxidative modifications, including S-sulfenylation (Cys-SOH), S-nitrosylation (Cys-SNO), and S-glutathionylation (Cys-SSG) (Figure 4A; Paulsen and Carroll, 2013). In addition, some cysteine residues in proteins are more redox-sensitive than others because of changes in the side chain orientation, charge, and altered exposure to ROS, affecting the efficiency of modification. For example, PTEN, a lipid phosphatase that antagonizes class I PI3K signaling by dephosphorylation of PI(3,4,5)P_3_, has a redox-sensitive cysteine residue in its catalytic center, which undergoes S-sulfenylation, leading to PTEN inactivation and increased class I PI3K signaling (Lee et al., 2002; Leslie et al., 2003; Zhang et al., 2017). The RAS GTPases are also regulated by cysteine oxidation, with the history of the RAS cysteine oxidation research tracked back to 1995 (Figure 3).

**FIGURE 5:**
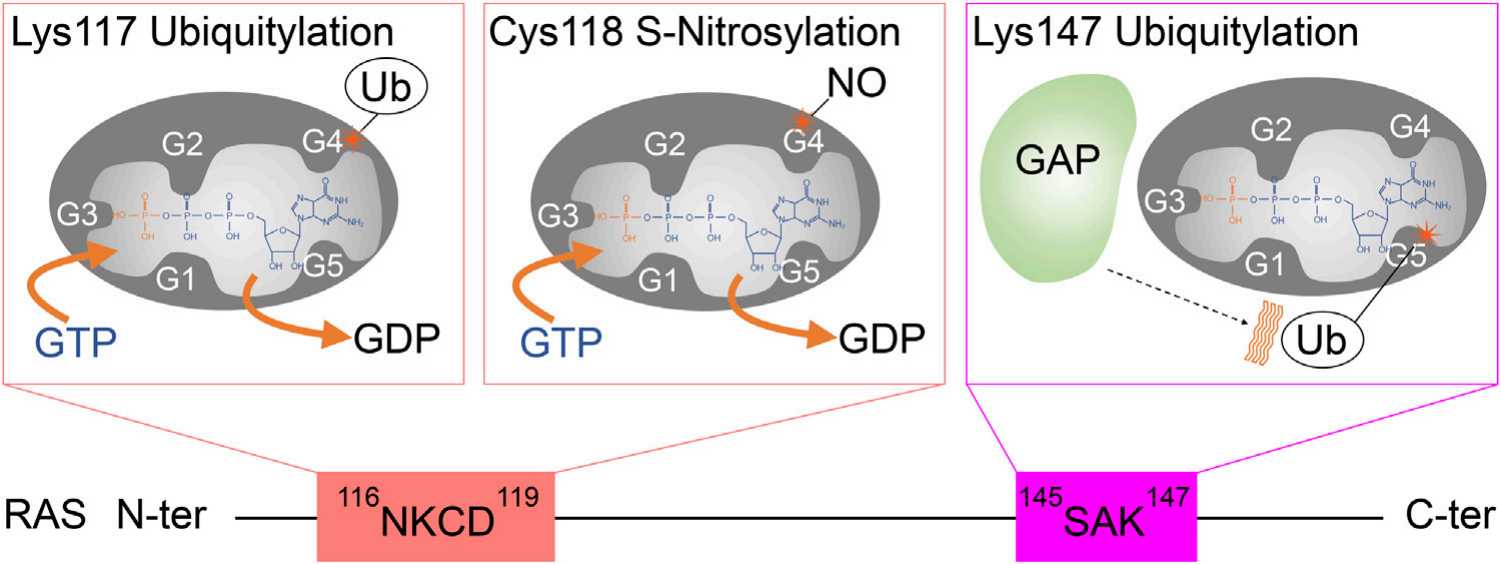
A schematic diagram highlighting the role of G4 and G5 post-translational modifications in RAS activation. Monoubiquitylation of RAS at Lys117, as well as S-nitrosylation of RAS at Cys118, increases GDP dissociation, leading to an increased GTP/GDP exchange rate. In contrast, monoubiquitylation of RAS at Lys147 impedes GAP-mediated GTP hydrolysis, which populates the active RAS GTP-bound “ON” state.

Novogrodsky’s group at the Tel Aviv University found that treatment of RAS with a variety of oxidative reagents, including hydrogen peroxide (H_2_O_2_), hemin, Hg^2+^, and NO, increases cellular RAS activity (Lander et al., 1995). Further, Cys118 was identified as the primary S-nitrosylation site in H-RAS. Cys118 is the most exposed solvent-accessible cysteine amongst three cysteine residues within the G-domain (Lander et al., 1996). Biochemical and structural studies of Cys118-nitrosylated H-RAS and a redox insensitive H-RAS variant (C118S) revealed that neither nitrosylation at this solvent-exposed site or mutation perturbs RAS structure, nucleotide cycling, or association with the RAS binding domain of CRAF (Mott et al., 1997; Williams et al., 2003). Subsequent functional analysis revealed that treatment with S-nitrosocysteine (CysNO), an NO donor, increases the GDP dissociation rate by ∼200-fold, resulting in the increased guanine nucleotide exchange rate, in the absence of a GEF (Williams et al., 2003; Heo and Campbell, 2004; Heo et al., 2005; Figure 5). Biochemical analysis revealed that various oxidants (e.g., superoxide, CysNO), but not H_2_O_2_, which produce a Cys118 thiol radical intermediate, can cause oxidation of the guanine nucleotide and destabilize guanine nucleotide-binding (Heo and Campbell, 2005), leading to enhanced guanine nucleotide exchange.

### Conservation of Cys118 Within RAS Superfamily Members

**FIGURE 6:**
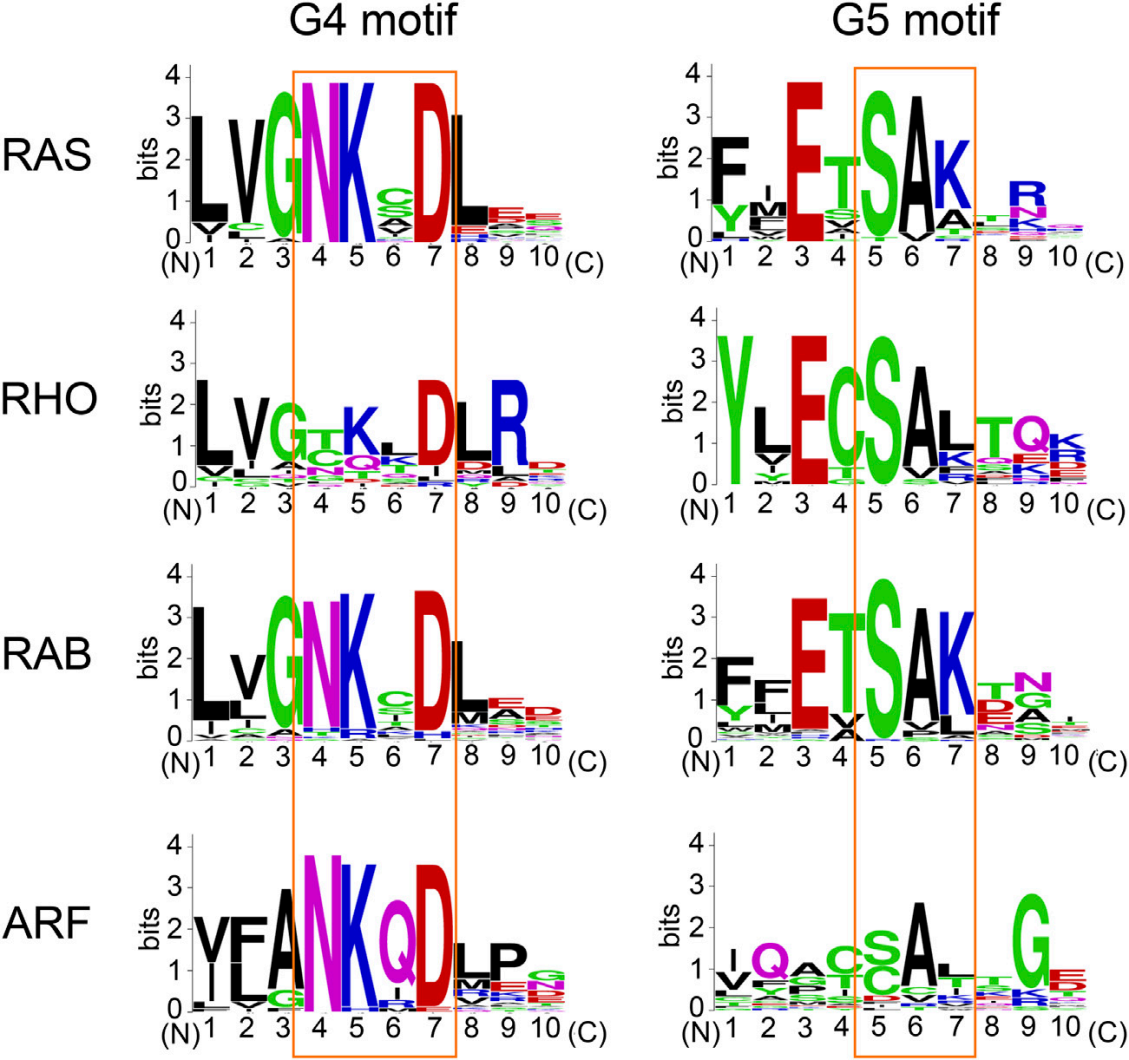
The conservation of amino acids within the G4 and G5 motifs. Sequence alignment performed using Clustal Omega (https://www.ebi.ac.uk/Tools/msa/clustalo/). The amino acid sequence logo for the G4 and G5 motifs was created using WebLogo (https://weblogo.berkeley.edu/logo.cgi).

About 20% of small GTPases possess a cysteine residue at the position equivalent to Cys118 in the RAS superfamily. Within the RAS and RAB sub-classes, 25 and 30% of these retain the Cys118 (RAS isotypes numbering) (Figure 6; Wennerberg et al., 2005), respectively. Similar to H-RAS, a RAS sub-class member RAP1A and a RAB sub-class member RAB3 undergo cysteine S-nitrosylation at the cysteine residue in the G4 motif, leading to enhanced guanine nucleotide exchange resulting in elevated RAS activity (Heo and Campbell, 2005; Heo et al., 2005). Thus, the role of Cys118 oxidation in regulation of GTPase activity appears to be conserved in several RAS and RAB sub-class GTPases, and possibly in the other small GTPases with the cysteine residue equivalent to RAS Cys118 (Raines et al., 2007; Lim et al., 2008; Davis et al., 2011; Mitchell et al., 2013).

### Ubiquitylation of Lys117 in G4 Motif

Lysine is a positively charged amino acid containing a long aliphatic sidechain and can undergo several post-translational modifications, such as acetylation, methylation, and ubiquitylation (Figure 4B). Ubiquitylation is a large lysine PTM, in which the 76 amino acid residue protein ubiquitin is conjugated to the ε-amine of the lysine residue in the target protein through an isopeptide bond formation to its carboxyl group of C-terminal glycine. The conjugated ubiquitin can be further polyubiquitylated. Lys48-linked polyubiquitylation induces proteasome-dependent protein degradation (Heride et al., 2014). This process typically requires four or more polyubiquitin chains (Thrower et al., 2000; Miller and Gordon, 2005). Protein monoubiquitylation, on the other hand, does not promote protein degradation but regulates other cell functions such as endocytic trafficking (Haglund et al., 2003; Mosesson et al., 2003) and DNA damage response (Uckelmann and Sixma, 2017).

In 2011, RAS was identified as a target for monoubiquitylation (Figure 3; Sasaki et al., 2011). Cell biology experiments conducted in HEK293T cells determined that both H- and K-RAS are targets for monoubiquitylation. Monoubiquitylation of H- and K-RAS appeared to promote RAS activation, as the ubiquitylated RAS were more populated in GTP-bound “ON” state and showed enhanced association with RAS effectors compared to the non-modified RAS. These findings suggest that the monoubiquitylation of RAS is linked to RAS activation (Sasaki et al., 2011). Tandem affinity purification of ubiquitylated H- and K-RAS4B (hereafter K-RAS) followed by mass spectrometry analysis identified Lys117 and Lys147 as major sites for monoubiquitylation, respectively. NMR analysis and cell biology experiments showed that monoubiquitylation of Lys117 stimulates nucleotide exchange in the absence of RAS GEF and thereby induces GTP loading and RAS activation (Baker et al., 2013b; Figure 5).

### Conservation of Lys117 Within RAS Superfamily Members

The lysine residue within the “N-K-X-D” G4 motif is highly conserved within the RAS superfamily (Figure 6). Within the RAS, RAB, and ARF sub-classes, almost all of these retain Lys117 (RAS isotypes numbering), while a few exceptions exist within the RHO sub-class GTPases (e.g., CDC42, TCL, RHOH) (Wennerberg et al., 2005). Furthermore, the lysine residue within the G4 motif is also highly conserved within the other G-protein families (Dever et al., 1987). Hence, it is considered that the GEF-independent activation via Lys117 monoubiquitylation may be a fundamental mechanism to regulate the activity of small GTPases and perhaps the other G-proteins as well.

## PTM Within RAS G5 Motif (^145^SAK^147^)

### Ubiquitylation of RAS Lys147 in the G5 Motif

Lys147 monoubiquitylation upregulates RAS activity in a manner distinct from Lys117 monoubiquitylation (Figure 5). Lys147 lies outside the Switch regions (Figures 1A,C). Using ubiquitin-conjugated K-RAS, our group discovered that Lys147 monoubiquitylation alters conformational dynamics of the Switch I and II regions and interferes with association of and downregulation by RAS GAPs while slightly altering GEF-dependent GDP/GTP exchange (Baker et al., 2013a; Figure 5). Biochemical, NMR, and computational analyses indicated that ubiquitin makes dynamic non-specific contacts with RAS, yet since the modification is large (∼8 kDa), it alters the conformation of Switch regions and dynamics of RAS structure (Baker et al., 2013a; Hobbs et al., 2013). This, in turn, alters recognition by GAP and effector proteins. In particular, the Lys147 monoubiquitylation enhances the association with the specific K-RAS effectors: CRAF, BRAF, and class I PI3K in HEK293T cells, while binding affinity appears unaffected with other effectors, such as phospholipase C (PLC) and calmodulin. These findings revealed a new function for ubiquitylation in modulating signaling through specific downstream pathways (Sasaki et al., 2011). While Lys147 monoubiquitylation of GDP-bound K-RAS significantly enhances the affinity to CRAF (more than 40-fold), monoubiquitylated GTP-bound K-RAS shows attenuated binding affinity for the RAS binding domain of certain RAS effectors (CRAF, RALGDS, and PI3Ks) (Thurman et al., 2017). These results suggest that monoubiquitylation in K-RAS Lys147 facilitates RAF association and promotes signaling in a GTP-independent manner. Also, further analysis showed that the linker length (at least seven to eight residues) and protein ligation size of ubiquitin are critical for the GAP defect (Hobbs et al., 2013).

Consistent with these results, cell biological analysis indicated that Lys147 monoubiquitylation promotes GTP loading of K-RAS. In mouse xenograft assays, a K-RAS G12V/K147L double mutant that cannot be ubiquitylated showed significantly decreased tumor mass and volume, compared to oncogenic K-RAS G12V expressing isogenic control cells, suggesting a critical role of Lys147 monoubiquitylation, or possibly through other modifications (e.g., acetylation, methylation), in tumor progression (Sasaki et al., 2011).

### Acetylation of RAS Lys147 in the G5 Motif

Lysine acetylation is a prevalent post-translational modification in eukaryotes and bacteria, and is mediated by the transfer of an acetyl CoA acetyl group by a cognate lysine acetyltransferase (Ali et al., 2018; Nakayasu et al., 2017). Acetylation of lysine decreases the overall positive charge of lysine residues and can create a docking site for other proteins (Figure 4B). Beyond its well-characterized role in regulating gene transcription through histone modification, lysine acetylation regulates diverse cellular processes through non-histone proteins (Ali et al., 2018).

Recent studies have shown that Lys147 in K-RAS also undergoes acetylation (Knyphausen et al., 2016; Song et al., 2016). The K-RAS K147Q mutation, which was generated to mimic Lys147-acetylation, increased the rate of guanine nucleotide exchange approximately three-fold higher than wild-type K-RAS (Song et al., 2016), which implies that acetylation of Lys147 in K-RAS may be involved in regulating guanine nucleotide exchange. However, the K147Q mutant may not mimic lysine acetylation as substitution of Lys147 with glutamine may disrupt a key interaction(s) important for guanine nucleotide-binding. Indeed, it has been shown that Lys147 acetylation did not affect the intrinsic and the GEF-dependent guanine nucleotide exchange (Knyphausen et al., 2016). Further studies are warranted to define the role of Lys147 acetylation in K-RAS functions.

### Methylation of RAS Lys147 in the G5 Motif

Protein methylation also occurs on side chain nitrogen atoms of lysine, arginine, and histidine residues. In contrast to the long-studied lysine acetylation, the roles of lysine-methylations beyond chromatin regulation are less well characterized, despite its earlier discovery in *Salmonella typhimurium* flagellin protein in 1959 (Ambler and Rees, 1959). Lysine modifications are more diverse than acetylation and can involve the transfer of one, two, or three methyl groups to the ε-amine of a lysine side chain through the conjugation of a methyl group from S-adenosyl methionine (SAM) by a lysine methyltransferase (Figure 4B). Unlike ubiquitylation and acetylation, lysine methylation maintains its overall positive charge. It is thus believed that the major function of lysine methylation is to provide a docking site for the proteins that recognize and bind methylated lysine (e.g., MBT and Tudor domains) (Lanouette et al., 2014; Teske and Hadden, 2017).

In 2019, mass spectrometry analysis of the immunoprecipitated endogenous RAS identified dimethylation at Lys5, adjacent to the G1 motif, as well as monomethylation at Lys147 in H-RAS (Figure 3) (Yoshino et al., 2019). While it is currently unclear whether Lys5 dimethylation is specific for all RAS isotypes, Lys147 is unique to the H-RAS. Given that substitutions at Lys147 to alanine, cysteine, or leucine do not significantly alter RAS activity (Sasaki et al., 2011; Baker et al., 2013a), it has been speculated that methylation of Lys147 does not alter RAS structure and that methylation of Lys147 may affect the H-RAS function by creating a docking site or blocking other PTMs. It is worth noting that methylation can prevent protein degradation by antagonizing ubiquitylation at the same targeted lysine residue (Lanouette et al., 2014); in yeast, 43% of methylated lysine residues are predicted to undergo ubiquitylation as well (Pang et al., 2010). Given that Lys147 in K-RAS undergoes monoubiquitylation, Lys147 methylation may negatively regulate RAS activation and monoubiquitylation-mediated effector switching.

### Conservation of Lys147 Within RAS Superfamily Members

The lysine residue within the “S-A-K” G5 motif is conserved in about 45% of RAS superfamily members (Figure 6; Wennerberg et al., 2005). The adjacent serine and alanine residues within the G5 motif are also highly conserved in each sub-class (Figure 6). Thus, the PTM of Lys147 (RAS isotypes numbering) may not be limited to RAS but present in other RAS superfamily GTPases. The G5 motif within some of the RHO, RAB, and ARF sub-classes contain “S-A-L,” “S-A-T,” “C-A-L,” and “C-A-T” sequences (Figure 6), and may undergo different PTMs within the G5 motif (e.g., phosphorylation at threonine residue of “S-A-T” motif and S-oxidation or S-nitrosylation at cysteine residue of “C-A-L” motif). Of note, the G5 motif is absent in several other G-proteins (e.g., heterotrimeric G-proteins and elongation factors). Whether the diverse sequences associated with the G5 motif in comparison to the more conserved G4 motif contribute to the functional difference of these RAS sub-classes remains unknown.

## Potential Therapeutic Application

### Oncogenic RAS Specific Inhibitors

Although RAS has been considered “undruggable” (Gysin et al., 2011; Samatar and Poulikakos, 2014; Stephen et al., 2014; Papke and Der, 2017; Welsch et al., 2017), recent discoveries identified covalent inhibitors that target Cys12 which is the reactive cysteine within the K-RAS G12C oncogenic mutant by designed peptide mimetics (Ostrem et al., 2013; Yoo et al., 2020). These inhibitors are shown to suppress tumor progression (Lito et al., 2016; Janes et al., 2018). Recently, Sotorasib, a K-RAS G12C inhibitor, has been granted accelerated approval by the Food and Drug Administration (FDA) (Canon et al., 2019; Hong et al., 2020) for the treatment of non-small-cell lung cancer (NSCLC). In addition, other K-RAS G12C inhibitors are now in multiple clinical trials, including phase II and phase III studies (Clinical Trial number: NCT04613596; NCT04685135; NCT04793958; NCT04449874; NCT04699188) (Hallin et al., 2020). While most K-RAS mutations occur at codon 12 (e.g., G12V, G12D), G12C is only one of the mutations that can lead to oncogenic RAS activation at this position. Hence, there is a need to develop therapeutics effective for other RAS mutant-driven cancers.

### Targeting the Enzymes Responsible for RAS PTMs

**FIGURE 7:**
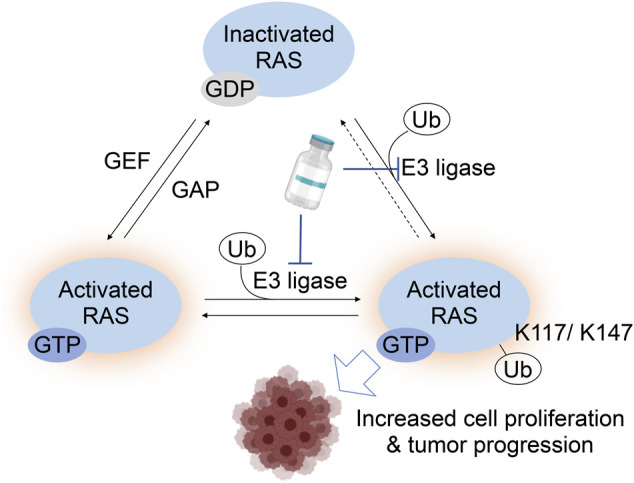
A RAS activation model by monoubiquitylation at Lys117 and Lys147. For RAS Lys117 ubiquitylation, a current working model is that Lys117 monoubiquitylation increase GTP-bound RAS by increased GTP/GDP exchange. The Lys147 monoubiquitylation inhibits the RAS GAP binding to RAS, which leads to the increased GTP-bound RAS. Hence, targeting the E3 ligase responsible for Lys117 and/or Lys147 monoubiquitylation is expected to suppress the RAS activation and possibly decrease tumorigenic activity or progression of cancer.

Given that the post-translational modifications identified in the G4 and G5 motifs are mediated by enzymes, we postulate that further mechanistic understanding of RAS regulation by PTMs of G4 and G5 motifs may unveil new approaches to suppress the RAS oncogenic activity that targets these modification enzymes (Figure 7). While the enzymes involved in RAS methylation remain unclear, several enzymes for RAS ubiquitylation and acetylation have been identified. Lysine deacetylases, HDAC6 and SIRT2, are suggested to negatively regulate K-RAS acetylation in cancer cells (Yang et al., 2013; Knyphausen et al., 2016). RABEX5, an E3 Ubiquitin ligase, catalyzes mono- and di-ubiquitylation of H- and N-RAS, but not K-RAS, which downregulates RAS activity (Xu et al., 2010; Yan et al., 2010; Washington et al., 2020). The ubiquitylation site(s) by RABEX5 remains unclear. A deubiquitinase OTUB1 has been identified as a negative regulator of RAS through a mammalian protein-protein interaction screening using H-RAS G12V mutant as the bait (Baietti et al., 2016). As Lys117 or Lys147 ubiquitylation upregulates RAS activity, it is unlikely that RABEX5 and OTUB1 modulate ubiquitylation of either Lys 117 or Lys147 in the G4 and G5 motifs. Hence, further studies exploring enzymes responsible for RAS ubiquitylation are required.

A promising new strategy to antagonize aberrant RAS signaling involves RAS degradation through ubiquitylation. These proteolysis-targeting chimera (PROTAC) approaches have proven to be an effective strategy for inhibiting specific protein targets (Churcher, 2017; Coleman and Crews, 2018). PROTACs induce proteolysis of a target protein by linking a target protein to the specific E3 ubiquitin ligase *via* a chemical tag (Khan et al., 2020). Importantly, PROTACs specifically targeting K-RAS or the K-RAS G12C mutant have recently been developed (Bery et al., 2020; Bond et al., 2020). Identifying RAS E3 ligases could aid in the application of PROTAC approaches for therapeutic inhibition of RAS as RAS-specific ligases may facilitate spatial/temporal localization needed for efficient RAS degradation. Clarifying which enzymes are responsible for RAS acetylation and methylation may provide another indirect way to suppress RAS activity by modulating these PTMs.

## Concluding Remarks

Post-translational modifications contribute to the diversification of protein function as well as the robustness to intra- and extracellular stress for maintaining cellular functions. Among the many post-translational modifications, S-oxygenation, S-nitrosylation, monoubiquitylation, acetylation, and methylation described in this review reflect reversible modifications that can modulate the function of RAS proteins. Divergent mechanisms involved in RAS activation through PTMs of the G4 and G5 motifs are likely to enable RAS to function at the distinctive subcellular localization, timing, and kinetics, apart from the canonical RAS regulatory pathway by GEFs and GAPs. Thus, RAS PTMs may play an important role in developing a new therapeutic approach for RAS-driven cancers. One of the next important steps will be to identify enzymes responsible for RAS PTMs as well as to clarify the physiological significance of these modifications in developmental processes, homeostasis, and disease states. PTMs associated with RAS G4 and G5 motifs may represent novel “Achille’s heels” for new anti-RAS approaches. Further understanding of these mechanisms might shed light on the development of effective therapeutic approaches.

## References

[B1] Ahearn I. Zhou M. Philips M. R. (2018). Posttranslational Modifications of RAS Proteins. Cold Spring Harb. Perspect. Med. 8, a031484. 10.1101/cshperspect.a031484 29311131 PMC6035883

[B2] Ali I. Conrad R. J. Verdin E. Ott M. (2018). Lysine Acetylation Goes Global: From Epigenetics to Metabolism and Therapeutics. Chem. Rev. 118, 1216–1252. 10.1021/acs.chemrev.7b00181 29405707 PMC6609103

[B3] Ambler R. P. Rees M. W. (1959). Ɛ-N-Methyl-lysine in Bacterial Flagellar Protein. Nature 184, 56–57. 10.1038/184056b0 13793118

[B4] Baietti M. F. Simicek M. Abbasi Asbagh L. Radaelli E. Lievens S. Crowther J. (2016). OTUB 1 Triggers Lung Cancer Development by Inhibiting RAS Monoubiquitination. EMBO Mol. Med. 8, 288–303. 10.15252/emmm.201505972 26881969 PMC4772950

[B5] Baker R. Lewis S. M. Sasaki A. T. Wilkerson E. M. Locasale J. W. Cantley L. C. (2013a). Site-specific Monoubiquitination Activates Ras by Impeding GTPase-Activating Protein Function. Nat. Struct. Mol. Biol. 20, 46–52. 10.1038/nsmb.2430 23178454 PMC3537887

[B6] Baker R. Wilkerson E. M. Sumita K. Isom D. G. Sasaki A. T. Dohlman H. G. (2013b). Differences in the Regulation of K-Ras and H-Ras Isoforms by Monoubiquitination. J. Biol. Chem. 288, 36856–36862. 10.1074/jbc.C113.525691 24247240 PMC3873545

[B7] Bery N. Miller A. Rabbitts T. (2020). A Potent KRAS Macromolecule Degrader Specifically Targeting Tumours with Mutant KRAS. Nat. Commun. 11, 3233. 10.1038/s41467-020-17022-w 32591521 PMC7319959

[B8] Bond M. J. Chu L. Nalawansha D. A. Li K. Crews C. M. (2020). Targeted Degradation of Oncogenic KRASG12C by VHL-Recruiting PROTACs. ACS Cent. Sci. 6, 1367–1375. 10.1021/acscentsci.0c00411 32875077 PMC7453568

[B9] Borrie S. C. Brems H. Legius E. Bagni C. (2017). Cognitive Dysfunctions in Intellectual Disabilities: The Contributions of the Ras-MAPK and PI3K-AKT-mTOR Pathways. Annu. Rev. Genom. Hum. Genet. 18, 115–142. 10.1146/annurev-genom-091416-035332 28859574

[B10] Bos J. L. (1989). Ras Oncogenes in Human Cancer: a Review. Cancer Res. 49, 4682–4689. 2547513

[B11] Bos J. L. Rehmann H. Wittinghofer A. (2007). GEFs and GAPs: Critical Elements in the Control of Small G Proteins. Cell 129, 865–877. 10.1016/j.cell.2007.05.018 17540168

[B12] Canon J. Rex K. Saiki A. Y. Mohr C. Cooke K. Bagal D. (2019). The Clinical KRAS(G12C) Inhibitor AMG 510 Drives Anti-tumour Immunity. Nature 575, 217–223. 10.1038/s41586-019-1694-1 31666701

[B13] Chang E. H. Gonda M. A. Ellis R. W. Scolnick E. M. Lowy D. R. (1982). Human Genome Contains Four Genes Homologous to Transforming Genes of Harvey and Kirsten Murine Sarcoma Viruses. Proc. Natl. Acad. Sci. 79, 4848–4852. 10.1073/pnas.79.16.4848 6289320 PMC346782

[B14] Cherfils J. Zeghouf M. (2013). Regulation of Small GTPases by GEFs, GAPs, and GDIs. Physiol. Rev. 93, 269–309. 10.1152/physrev.00003.2012 23303910

[B15] Churcher I. (2017). Protac-induced Protein Degradation in Drug Discovery: Breaking the Rules or Just Making New Ones? J. Med. Chem. 61, 444–452. 10.1021/acs.jmedchem.7b01272 29144739

[B16] Coleman K. G. Crews C. M. (2018). Proteolysis-targeting Chimeras: Harnessing the Ubiquitin-Proteasome System to Induce Degradation of Specific Target Proteins. Annu. Rev. Cancer Biol. 2, 41–58. 10.1146/annurev-cancerbio-030617-050430

[B17] Cool R. H. Schmidt G. Lenzen C. U. Prinz H. Vogt D. Wittinghofer A. (1999). The Ras Mutant D119N Is Both Dominant Negative and Activated. Mol. Cel. Biol. 19, 6297–6305. 10.1128/mcb.19.9.6297 PMC84598 10454576

[B18] Davies M. J. (2016). Protein Oxidation and Peroxidation. Biochem. J. 473, 805–825. 10.1042/BJ20151227 27026395 PMC4819570

[B19] Davis M. F. Vigil D. Campbell S. L. (2011). Regulation of Ras Proteins by Reactive Nitrogen Species. Free Radic. Biol. Med. 51, 565–575. 10.1016/j.freeradbiomed.2011.05.003 21616138 PMC3549334

[B20] Denayer E. Parret A. Chmara M. Schubbert S. Vogels A. Devriendt K. (2008). Mutation Analysis in Costello Syndrome: Functional and Structural Characterization of theHRASp.Lys117Arg Mutation. Hum. Mutat. 29, 232–239. 10.1002/humu.20616 17979197

[B21] Dever T. E. Glynias M. J. Merrick W. C. (1987). GTP-binding Domain: Three Consensus Sequence Elements with Distinct Spacing. Proc. Natl. Acad. Sci. 84, 1814–1818. 10.1073/pnas.84.7.1814 3104905 PMC304531

[B22] Downward J. (2003). Targeting RAS Signalling Pathways in Cancer Therapy. Nat. Rev. Cancer 3, 11–22. 10.1038/nrc969 12509763

[B23] Edkins S. O’Meara S. Parker A. Stevens C. Reis M. Jones S. (2006). Recurrent KRAS Codon 146 Mutations in Human Colorectal Cancer. Cancer. Biol. Ther. 5, 928–932. 10.4161/cbt.5.8.3251 16969076 PMC2714972

[B24] Feig L. A. Pan B. T. Roberts T. M. Cooper G. M. (1986). Isolation of Ras GTP-Binding Mutants Using an *In Situ* colony-binding Assay. Proc. Natl. Acad. Sci. 83, 4607–4611. 10.1073/pnas.83.13.4607 3088563 PMC323790

[B25] Feuerstein J. Goody R. S. Wittinghofer A. (1987). Preparation and Characterization of Nucleotide-free and Metal Ion-free P21 "apoprotein". J. Biol. Chem. 262, 8455–8458. 10.1016/s0021-9258(18)47433-9 3298232

[B26] Forbes S. A. Bindal N. Bamford S. Cole C. Kok C. Y. Beare D. (2010). COSMIC: Mining Complete Cancer Genomes in the Catalogue of Somatic Mutations in Cancer. Nucleic Acids Res. 39, D945–D950. 10.1093/nar/gkq929 20952405 PMC3013785

[B27] Ford B. Boykevisch S. Zhao C. Kunzelmann S. Bar-Sagi D. Herrmann C. (2009). Characterization of a Ras Mutant with Identical GDP- and GTP-Bound Structures,. Biochemistry 48, 11449–11457. 10.1021/bi901479b 19883123 PMC4160238

[B28] Gideon P. John J. Frech M. Lautwein A. Clark R. Scheffler J. E. (1992). Mutational and Kinetic Analyses of the GTPase-Activating Protein (GAP)-p21 Interaction: the C-Terminal Domain of GAP Is Not Sufficient for Full Activity. Mol. Cel. Biol. 12, 2050–2056. 10.1128/mcb.12.5.2050 PMC364376 1569940

[B29] Gray J. L. Delft F. Brennan P. E. (2020). Targeting the Small GTPase Superfamily through Their Regulatory Proteins. Angew. Chem. Int. Ed. 59, 6342–6366. 10.1002/anie.201900585 PMC7204875 30869179

[B30] Gremer L. Merbitz-Zahradnik T. Dvorsky R. Cirstea I. C. Kratz C. P. Zenker M. (2011). Germline KRAS Mutations Cause Aberrant Biochemical and Physical Properties Leading to Developmental Disorders. Hum. Mutat. 32, 33–43. 10.1002/humu.21377 20949621 PMC3117284

[B31] Gysin S. Salt M. Young A. McCormick F. (2011). Therapeutic Strategies for Targeting Ras Proteins. Genes & Cancer 2, 359–372. 10.1177/1947601911412376 21779505 PMC3128641

[B32] Haglund K. Di Fiore P. P. Dikic I. (2003). Distinct Monoubiquitin Signals in Receptor Endocytosis. Trends Biochem. Sci. 28, 598–604. 10.1016/j.tibs.2003.09.005 14607090

[B33] Hallin J. Engstrom L. D. Hargis L. Calinisan A. Aranda R. Briere D. M. (2020). The KRAS G12CInhibitor MRTX849 Provides Insight toward Therapeutic Susceptibility of KRAS-Mutant Cancers in Mouse Models and Patients. Cancer Dis. 10, 54–71. 10.1158/2159-8290.CD-19-1167 PMC6954325 31658955

[B34] Hennig A. Markwart R. Esparza-Franco M. A. Ladds G. Rubio I. (2015). Ras Activation Revisited: Role of GEF and GAP Systems. Biol. Chem. 396, 831–848. 10.1515/hsz-2014-0257 25781681

[B35] Heo J. Campbell S. L. (2004). Mechanism of p21RasS-Nitrosylation and Kinetics of Nitric Oxide-Mediated Guanine Nucleotide Exchange†. Biochemistry 43, 2314–2322. 10.1021/bi035275g 14979728

[B36] Heo J. Campbell S. L. (2005). Superoxide Anion Radical Modulates the Activity of Ras and Ras-Related GTPases by a Radical-Based Mechanism Similar to that of Nitric Oxide. J. Biol. Chem. 280, 12438–12445. 10.1074/jbc.M414282200 15684418

[B37] Heo J. Prutzman K. C. Mocanu V. Campbell S. L. (2005). Mechanism of Free Radical Nitric Oxide-Mediated Ras Guanine Nucleotide Dissociation. J. Mol. Biol. 346, 1423–1440. 10.1016/j.jmb.2004.12.050 15713491

[B38] Heride C. Urbé S. Clague M. J. (2014). Ubiquitin Code Assembly and Disassembly. Curr. Biol. 24, R215–R220. 10.1016/j.cub.2014.02.002 24650902

[B39] Herrmann C. Martin G. A. Wittinghofer A. (1995). Quantitative Analysis of the Complex between P21 and the Ras-Binding Domain of the Human Raf-1 Protein Kinase. J. Biol. Chem. 270, 2901–2905. 10.1074/jbc.270.7.2901 7852367

[B40] Hobbs G. A. Gunawardena H. P. Baker R. Chen X. Campbell S. L. (2013). Site-specific Monoubiquitination Activates Ras by Impeding GTPase-Activating Protein Function. Small GTPases 4, 186–192. 10.4161/sgtp.26270 24030601 PMC3976977

[B41] Hong D. S. Fakih M. G. Strickler J. H. Desai J. Durm G. A. Shapiro G. I. (2020). KRASG12C Inhibition with Sotorasib in Advanced Solid Tumors. N. Engl. J. Med. 383, 1207–1217. 10.1056/NEJMoa1917239 32955176 PMC7571518

[B42] Janakiraman M. Vakiani E. Zeng Z. Pratilas C. A. Taylor B. S. Chitale D. (2010). Genomic and Biological Characterization of Exon 4 KRAS Mutations in Human Cancer. Cancer Res. 70, 5901–5911. 10.1158/0008-5472.CAN-10-0192 20570890 PMC2943514

[B43] Janes M. R. Zhang J. Li L.-S. Hansen R. Peters U. Guo X. (2018). Targeting KRAS Mutant Cancers with a Covalent G12C-specific Inhibitor. Cell 172, 578–589. 10.1016/j.cell.2018.01.006 29373830

[B44] John J. Rensland H. Schlichting I. Vetter I. Borasio G. D. Goody R. S. (1993). Kinetic and Structural Analysis of the Mg(2+)-Binding Site of the Guanine Nucleotide-Binding Protein p21H-Ras. J. Biol. Chem. 268, 923–929. 10.1016/s0021-9258(18)54022-9 8419371

[B45] Karnoub A. E. Weinberg R. A. (2008). Ras Oncogenes: Split Personalities. Nat. Rev. Mol. Cel Biol. 9, 517–531. 10.1038/nrm2438 PMC3915522 18568040

[B46] Khan S. He Y. Zhang X. Yuan Y. Pu S. Kong Q. (2020). PROteolysis TArgeting Chimeras (PROTACs) as Emerging Anticancer Therapeutics. Oncogene 39, 4909–4924. 10.1038/s41388-020-1336-y 32475992 PMC7319888

[B47] Kiel C. Filchtinski D. Spoerner M. Schreiber G. Kalbitzer H. R. Herrmann C. (2009). Improved Binding of Raf to Ras·GDP Is Correlated with Biological Activity. J. Biol. Chem. 284, 31893–31902. 10.1074/jbc.M109.031153 19776012 PMC2797260

[B48] Kinoshita K. Sadanami K. Kidera A. Go N. (1999). Structural Motif of Phosphate-Binding Site Common to Various Protein Superfamilies: All-Against-All Structural Comparison of Protein-Mononucleotide Complexes. Protein Eng. 12, 11–14. 10.1093/protein/12.1.11 10065705

[B49] Knyphausen P. Lang F. Baldus L. Extra A. Lammers M. (2016). Insights into K-Ras 4B Regulation by post-translational Lysine Acetylation. Biol. Chem. 397, 1071–1085. 10.1515/hsz-2016-0118 27176741

[B50] Kötting C. Kallenbach A. Suveyzdis Y. Wittinghofer A. Gerwert K. (2008). The GAP Arginine finger Movement into the Catalytic Site of Ras Increases the Activation Entropy. Proc. Natl. Acad. Sci. 105, 6260–6265. 10.1073/pnas.0712095105 18434546 PMC2359817

[B51] Lander H. M. Milbank A. J. Tauras J. M. Hajjar D. P. Hempstead B. L. Schwartz G. D. (1996). Redox Regulation of Cell Signalling. Nature 381, 380–381. 10.1038/381380a0 8632794

[B52] Lander H. M. Ogiste J. S. Teng K. K. Novogrodsky A. (1995). p21 as a Common Signaling Target of Reactive Free Radicals and Cellular Redox Stress. J. Biol. Chem. 270, 21195–21198. 10.1074/jbc.270.36.21195 7673152

[B53] Lanouette S. Mongeon V. Figeys D. Couture J. F. (2014). The Functional Diversity of Protein Lysine Methylation. Mol. Syst. Biol. 10, 724. 10.1002/msb.134974 24714364 PMC4023394

[B54] Lee S.-R. Yang K.-S. Kwon J. Lee C. Jeong W. Rhee S. G. (2002). Reversible Inactivation of the Tumor Suppressor PTEN by H2O2. J. Biol. Chem. 277, 20336–20342. 10.1074/jbc.M111899200 11916965

[B55] Leslie N. R. Bennett D. Lindsay Y. E. Stewart H. Gray A. Downes C. P. (2003). Redox Regulation of PI 3-kinase Signalling via Inactivation of PTEN. EMBO J. 22, 5501–5510. 10.1093/emboj/cdg513 14532122 PMC213768

[B56] Li S. Balmain A. Counter C. M. (2018). A Model for RAS Mutation Patterns in Cancers: Finding the Sweet Spot. Nat. Rev. Cancer 18, 767–777. 10.1038/s41568-018-0076-6 30420765

[B57] Lim K.-H. Ancrile B. B. Kashatus D. F. Counter C. M. (2008). Tumour Maintenance Is Mediated by eNOS. Nature 452, 646–649. 10.1038/nature06778 18344980 PMC2688829

[B58] Lito P. Solomon M. Li L.-S. Hansen R. Rosen N. (2016). Allele-specific Inhibitors Inactivate Mutant KRAS G12C by a Trapping Mechanism. Science 351, 604–608. 10.1126/science.aad6204 26841430 PMC4955282

[B59] Malumbres M. Barbacid M. (2003). RAS Oncogenes: the First 30 Years. Nat. Rev. Cancer 3, 459–465. 10.1038/nrc1097 12778136

[B60] Miller J. Gordon C. (2005). The Regulation of Proteasome Degradation by Multi-Ubiquitin Chain Binding Proteins. FEBS Lett. 579, 3224–3230. 10.1016/j.febslet.2005.03.042 15943965

[B61] Mitchell L. Hobbs G. A. Aghajanian A. Campbell S. L. (2013). Redox Regulation of Ras and Rho GTPases: Mechanism and Function. Antioxid. Redox Signaling 18, 250–258. 10.1089/ars.2012.4687 PMC3518547 22657737

[B62] Moore A. R. Rosenberg S. C. McCormick F. Malek S. (2020). RAS-targeted Therapies: Is the Undruggable Drugged?. Nat. Rev. Drug Discov. 19, 533–552. 10.1038/s41573-020-0068-6 32528145 PMC7809886

[B63] Mosesson Y. Shtiegman K. Katz M. Zwang Y. Vereb G. Szollosi J. (2003). Endocytosis of Receptor Tyrosine Kinases Is Driven by Monoubiquitylation, Not Polyubiquitylation. J. Biol. Chem. 278, 21323–21326. 10.1074/jbc.C300096200 12719435

[B64] Mott H. R. Carpenter J. W. Campbell S. L. (1997). Structural and Functional Analysis of a Mutant Ras Protein that Is Insensitive to Nitric Oxide Activation†. Biochemistry 36, 3640–3644. 10.1021/bi962790o 9132016

[B65] Nakayasu E. S. Burnet M. C. Walukiewicz H. E. Wilkins C. S. Shukla A. K. Brooks S. (2017). Ancient Regulatory Role of Lysine Acetylation in Central Metabolism. mBio 8, 1395. 10.1128/mBio.01894-17 PMC5705920 29184018

[B66] Nassar A. H. Adib E. Kwiatkowski D. J. (2021). Distribution of KRASG12C Somatic Mutations across Race, Sex, and Cancer Type. N. Engl. J. Med. 384, 185–187. 10.1056/NEJMc2030638 33497555

[B67] Niihori T. Aoki Y. Okamoto N. Kurosawa K. Ohashi H. Mizuno S. (2011). HRAS Mutants Identified in Costello Syndrome Patients Can Induce Cellular Senescence: Possible Implications for the Pathogenesis of Costello Syndrome. J. Hum. Genet. 56, 707–715. 10.1038/jhg.2011.85 21850009

[B68] Ostrem J. M. Peters U. Sos M. L. Wells J. A. Shokat K. M. (2013). K-Ras(G12C) Inhibitors Allosterically Control GTP Affinity and Effector Interactions. Nature 503, 548–551. 10.1038/nature12796 24256730 PMC4274051

[B69] Pai E. F. Kabsch W. Krengel U. Holmes K. C. John J. Wittinghofer A. (1989). Structure of the Guanine-Nucleotide-Binding Domain of the Ha-Ras Oncogene Product P21 in the Triphosphate Conformation. Nature 341, 209–214. 10.1038/341209a0 2476675

[B70] Pang C. Gasteiger E. Wilkins M. R. (2010). Identification of Arginine- and Lysine-Methylation in the Proteome of *Saccharomyces cerevisiae* and its Functional Implications. BMC Genomics 11, 92. 10.1186/1471-2164-11-92 20137074 PMC2830191

[B71] Papke B. Der C. J. (2017). Drugging RAS: Know the Enemy. Science 355, 1158–1163. 10.1126/science.aam7622 28302824

[B72] Paulsen C. E. Carroll K. S. (2013). Cysteine-Mediated Redox Signaling: Chemistry, Biology, and Tools for Discovery. Chem. Rev. 113, 4633–4679. 10.1021/cr300163e 23514336 PMC4303468

[B73] Prior I. A. Hood F. E. Hartley J. L. (2020). The Frequency of Ras Mutations in Cancer. Cancer Res. 80, 2969–2974. 10.1158/0008-5472.CAN-19-3682 32209560 PMC7367715

[B74] Prior I. A. Lewis P. D. Mattos C. (2012). A Comprehensive Survey of Ras Mutations in Cancer. Cancer Res. 72, 2457–2467. 10.1158/0008-5472.CAN-11-2612 22589270 PMC3354961

[B75] Pylayeva-Gupta Y. Grabocka E. Bar-Sagi D. (2011). RAS Oncogenes: Weaving a Tumorigenic Web. Nat. Rev. Cancer 11, 761–774. 10.1038/nrc3106 21993244 PMC3632399

[B76] Raines K. Bonini M. Campbell S. (2007). Nitric Oxide Cell Signaling: S-Nitrosation of Ras Superfamily GTPases. Cardiovasc. Res. 75, 229–239. 10.1016/j.cardiores.2007.04.013 17559822

[B77] Ratner N. Miller S. J. (2015). A RASopathy Gene Commonly Mutated in Cancer: the Neurofibromatosis Type 1 Tumour Suppressor. Nat. Rev. Cancer 15, 290–301. 10.1038/nrc3911 25877329 PMC4822336

[B78] Rauen K. A. (2013). The RASopathies. Annu. Rev. Genom. Hum. Genet. 14, 355–369. 10.1146/annurev-genom-091212-153523 PMC4115674 23875798

[B79] Samatar A. A. Poulikakos P. I. (2014). Targeting RAS-ERK Signalling in Cancer: Promises and Challenges. Nat. Rev. Drug Discov. 13, 928–942. 10.1038/nrd4281 25435214

[B80] Sasaki A. T. Carracedo A. Locasale J. W. Anastasiou D. Takeuchi K. Kahoud E. R. (2011). Ubiquitination of K-Ras Enhances Activation and Facilitates Binding to Select Downstream Effectors. Sci. Signaling 4, ra13. 10.1126/scisignal.2001518 PMC3437993 21386094

[B81] Scheffzek K. Ahmadian M. R. Kabsch W. Wiesmüller L. Lautwein A. Schmitz F. (1997). The Ras-RasGAP Complex: Structural Basis for GTPase Activation and its Loss in Oncogenic Ras Mutants. Science 277, 333–338. 10.1126/science.277.5324.333 9219684

[B82] Simanshu D. K. Nissley D. V. McCormick F. (2017). RAS Proteins and Their Regulators in Human Disease. Cell 170, 17–33. 10.1016/j.cell.2017.06.009 28666118 PMC5555610

[B83] Smith G. Bounds R. Wolf H. Steele R. J. C. Carey F. A. Wolf C. R. (2010). Activating K-Ras Mutations Outwith 'hotspot' Codons in Sporadic Colorectal Tumours - Implications for Personalised Cancer Medicine. Br. J. Cancer 102, 693–703. 10.1038/sj.bjc.6605534 20147967 PMC2837563

[B84] Smith M. J. Neel B. G. Ikura M. (2013). NMR-based Functional Profiling of RASopathies and Oncogenic RAS Mutations. Proc. Natl. Acad. Sci. 110, 4574–4579. 10.1073/pnas.1218173110 23487764 PMC3607025

[B85] Song H. Y. Biancucci M. Kang H.-J. O'Callaghan C. Park S.-H. Principe D. R. (2016). SIRT2 Deletion Enhances KRAS-Induced Tumorigenesis *In Vivo* by Regulating K147 Acetylation Status. Oncotarget 7, 80336–80349. 10.18632/oncotarget.12015 27637077 PMC5340253

[B86] Stalnecker C. A. Der C. J. (2020). RAS, Wanted Dead or Alive: Advances in Targeting RAS Mutant Cancers. Sci. Signal. 13, eaay6013. 10.1126/scisignal.aay6013 32209699 PMC7393681

[B87] Stephen A. G. Esposito D. Bagni R. K. McCormick F. (2014). Dragging Ras Back in the Ring. Cancer Cell 25, 272–281. 10.1016/j.ccr.2014.02.017 24651010

[B88] Teske K. A. Hadden M. K. (2017). Methyllysine Binding Domains: Structural Insight and Small Molecule Probe Development. Eur. J. Med. Chem. 136, 14–35. 10.1016/j.ejmech.2017.04.047 28478342

[B89] Thrower J. S. Hoffman L. Rechsteiner M. Pickart C. M. (2000). Recognition of the Polyubiquitin Proteolytic Signal. EMBO J. 19, 94–102. 10.1093/emboj/19.1.94 10619848 PMC1171781

[B90] Thurman R. Siraliev-Perez E. Campbell S. L. (2020). RAS Ubiquitylation Modulates Effector Interactions. Small GTPases 11, 180–185. 10.1080/21541248.2017.1371267 29185849 PMC7549706

[B91] Tidyman W. E. Rauen K. A. (2009). The RASopathies: Developmental Syndromes of Ras/MAPK Pathway Dysregulation. Curr. Opin. Genet. Develop. 19, 230–236. 10.1016/j.gde.2009.04.001 PMC2743116 19467855

[B92] Traut T. W. (1994). Physiological Concentrations of Purines and Pyrimidines. Mol. Cel. Biochem. 140, 1–22. 10.1007/BF00928361 7877593

[B93] Uckelmann M. Sixma T. K. (2017). Histone Ubiquitination in the DNA Damage Response. DNA Repair 56, 92–101. 10.1016/j.dnarep.2017.06.011 28624371

[B94] Vetter I. R. Wittinghofer A. (2001). The Guanine Nucleotide-Binding Switch in Three Dimensions. Science 294, 1299–1304. 10.1126/science.1062023 11701921

[B95] Vigil D. Cherfils J. Rossman K. L. Der C. J. (2010). Ras Superfamily GEFs and GAPs: Validated and Tractable Targets for Cancer Therapy? Nat. Rev. Cancer 10, 842–857. 10.1038/nrc2960 21102635 PMC3124093

[B96] Wallace A. C. Laskowski R. A. Thornton J. M. (1995). LIGPLOT: a Program to Generate Schematic Diagrams of Protein-Ligand Interactions. Protein Eng. Des. Sel 8, 127–134. 10.1093/protein/8.2.127 7630882

[B97] Washington C. Chernet R. Gokhale R. H. Martino-Cortez Y. Liu H.-Y. Rosenberg A. M. (2020). A Conserved, N-Terminal Tyrosine Signal Directs Ras for Inhibition by Rabex-5. Plos Genet. 16, e1008715. 10.1371/journal.pgen.1008715 32559233 PMC7329146

[B98] Welsch M. E. Kaplan A. Chambers J. M. Stokes M. E. Bos P. H. Zask A. (2017). Multivalent Small-Molecule Pan-RAS Inhibitors. Cell 168, 878–889. 10.1016/j.cell.2017.02.006 28235199 PMC5362268

[B99] Wennerberg K. Rossman K. L. Der C. J. (2005). The Ras Superfamily at a Glance. J. Cel Sci. 118, 843–846. 10.1242/jcs.01660 15731001

[B100] Williams J. G. Pappu K. Campbell S. L. (2003). Structural and Biochemical Studies of p21Ras S-Nitrosylation and Nitric Oxide-Mediated Guanine Nucleotide Exchange. Proc. Natl. Acad. Sci. 100, 6376–6381. 10.1073/pnas.1037299100 12740440 PMC164454

[B101] Wittinghofer A. Vetter I. R. (2011). Structure-function Relationships of the G Domain, a Canonical Switch Motif. Annu. Rev. Biochem. 80, 943–971. 10.1146/annurev-biochem-062708-134043 21675921

[B102] Wójcik P. Kulig J. Okoń K. Zazula M. Moździoch I. Niepsuj A. (2008). KRAS Mutation Profile in Colorectal Carcinoma and Novel Mutation-Iinternal Tandem Duplication in KRAS. Pol. J. Pathol. 59, 93–96. 18669174

[B103] Xu L. Lubkov V. Taylor L. J. Bar-Sagi D. (2010). Feedback Regulation of Ras Signaling by Rabex-5-Mediated Ubiquitination. Curr. Biol. 20, 1372–1377. 10.1016/j.cub.2010.06.051 20655225 PMC3436604

[B104] Yan H. Jahanshahi M. Horvath E. A. Liu H-Y. Pfleger C. M. (2010). Rabex-5 Ubiquitin Ligase Activity Restricts Ras Signaling to Establish Pathway Homeostasis in Drosophila. Curr. Biol. 20, 1378–1382. 10.1016/j.cub.2010.06.058 20655224 PMC2938185

[B105] Yang M. H. Laurent G. Bause A. S. Spang R. German N. Haigis M. C. (2013). HDAC6 and SIRT2 Regulate the Acetylation State and Oncogenic Activity of Mutant K-RAS. Mol. Cancer Res. 11, 1072–1077. 10.1158/1541-7786.MCR-13-0040-T 23723075 PMC3778089

[B106] Yoo D. Y. Hauser A. D. Joy S. T. Bar-Sagi D. Arora P. S. (2020). Covalent Targeting of Ras G12C by Rationally Designed Peptidomimetics. ACS Chem. Biol. 15, 1604–1612. 10.1021/acschembio.0c00204 32378881 PMC7739374

[B107] Yoshino H. Yin G. Kawaguchi R. Popov K. I. Temple B. Sasaki M. (2019). Identification of Lysine Methylation in the Core GTPase Domain by GoMADScan. PLoS One 14, e0219436–17. 10.1371/journal.pone.0219436 31390367 PMC6685615

[B108] Zhang Y. Han S.-J. Park I. Kim I. Chay K.-O. Kim S. (2017). Redox Regulation of the Tumor Suppressor PTEN by Hydrogen Peroxide and Tert-Butyl Hydroperoxide. Int. J. Mol. Sci. 18, 982–. 10.3390/ijms18050982 PMC5454895 28489026

